# Mapping and appraising evidence syntheses of resuscitation training for healthcare professionals: Protocol for a systematic review of reviews

**DOI:** 10.1371/journal.pone.0349693

**Published:** 2026-07-13

**Authors:** Defi Efendi, Chiyar Edison Sunarya, Mawardi Ihsan, Hazrina Adelia, Nana Rochana, Heni Ekawati, Sholihatul Amaliya, Cho Lee Wong, on behalf of the LIFESAVER (Leading Innovations for Emergency Support and Vital Enhancements in Resuscitation) Study Network

**Affiliations:** 1 Department of Pediatric Nursing, Faculty of Nursing, Universitas Indonesia, West Java, Indonesia; 2 Neonatal Intensive Care Unit, Universitas Indonesia Hospital, West Java, Indonesia; 3 Department of Medical-Surgical Nursing, Faculty of Nursing, Universitas Indonesia, West Java, Indonesia; 4 Department of Pharmacology and Clinical Pharmacy, Faculty of Pharmacy, Universitas Gadjah Mada, Yogyakarta, Indonesia; 5 Faculty of Pharmacy and Health Sciences, Abdurrab University, Riau, Indonesia; 6 Department of Nursing, Faculty of Medicine, Universitas Diponegoro, Semarang, Indonesia; 7 Department of Pediatric Nursing, Universitas Noor Huda Mustofa, Bangkalan, East Java, Indonesia; 8 Department of Nursing-Faculty of Health, Universitas Brawijaya, Malang, Indonesia; 9 The Nethersole School of Nursing, The Chinese University of Hong Kong, Ma Liu Shui, Hong Kong; Athens Medical Group, Psychiko Clinic, GREECE

## Abstract

**Background:**

The number of systematic reviews on resuscitation training has grown rapidly, offering valuable evidence to guide healthcare decision-making. However, methodological shortcomings can compromise validity and yield biased or misleading conclusions. This systematic review aims to systematically map the distribution of published systematic reviews on resuscitation training interventions, evaluate their methodological quality, investigate contextual and methodological factors associated with systematic review quality, and delineate priorities for future systematic reviews.

**Methods:**

This systematic review protocol follows the PRISMA-P guidelines. A comprehensive search from the inception to August 2025 will be conducted across eight major databases, comprising CENTRAL, EMBASE, MEDLINE, ProQuest, Scopus, Web of Science, CINAHL, and ERIC, to identify published reviews on resuscitation training interventions for healthcare professionals and students, excluding laypersons. All reviews that are appropriate for the inclusion criteria will be screened and selected independently by two reviewers, with disagreements resolved by a third reviewer. Data extraction will cover study characteristics and methodological features, and quality appraisal will be performed using the AMSTAR 2 tool. Findings will be synthesized narratively following the SWiM framework, focusing on methodological rigor and evidence gaps.

**Conclusion:**

This study will systematically evaluate the methodological quality of published systematic reviews on resuscitation training using the AMSTAR 2 tool, providing key insights to promote higher standards in systematic review conduct and reporting within healthcare education.

## Introduction

Resuscitation training is a critical component of emergency care systems, directly impacting survival rates and clinical outcomes through the quality of resuscitation efforts [1,2]. Evidence shows that cardiopulmonary resuscitations (CPR) increase global survival rates of out-of-hospital cardiac arrest (OHCA) from around 1% to 5% in the past 40 years [1]. In recent years, there has been a significant increase in the literature on evaluating resuscitation training interventions. This increase is also accompanied by a growing number of systematic reviews synthesizing the evidence [3–5]. These reviews have explored diverse trainee populations, types of resuscitation training, delivery methods, and outcomes [6–9].

High-quality systematic reviews and meta-analyses (SR-MA) are essential tools for decision-makers and researchers, providing a rapid understanding of the latest developments in a research problem [10]. Nevertheless, low-quality systematic reviews warrant caution. A review with an inadequate search process may fail to gather comprehensive evidence, leading to selection bias. Another example is a review that based the conclusions solely on p-value, without considering the quality of evidence for specific outcomes. All these can affect the review’s conclusions and lead to clinical implications being overstated or understated, which may not accurately reflect reality [11,12]. These cases underscore the necessity for rigorously conducted and reported systematic reviews to ensure that synthesized evidence accurately informs resuscitation training practices.

Various guidelines have been developed to assist researchers in conducting systematic reviews and meta-analyses. These guidelines include the Preferred Reporting Items for Systematic Reviews and Meta-Analyses (PRISMA), the Cochrane Handbook, the Quality of Reporting of Meta-analyses (QUORUM), and the Meta-analyses Of Observational Studies in Epidemiology (MOOSE) [13–16]. Furthermore, several appraisal tools have also been designed to help researchers assess these studies. The Measurement Tool to Assess Systematic Reviews (AMSTAR, including its revised and updated version—R-AMSTAR and AMSTAR 2), the Scottish Intercollegiate Guidelines Network (SIGN) checklist, and the Risk of Bias Assessment Tool for Systematic Reviews (ROBIS) serve as their examples [17–21].

The AMSTAR 2 (A MeaSurement Tool to Assess Systematic Reviews) is a widely used, valid, and reliable instrument for evaluating the methodological quality of SR-MA [22]. Developed as an update to the original AMSTAR, it incorporates criteria tailored to the increasing complexity of modern reviews, including those that synthesize non-randomized studies—an aspect particularly relevant to resuscitation training studies. By providing a standardized framework for appraisal, AMSTAR 2 enhances the transparency and consistency of review assessments. This enhancement, in turn, enables researchers, clinicians, and policymakers to assess the reliability and applicability of a review’s findings, thereby supporting evidence-based decision-making in healthcare [23].

To strengthen the rigor of evidence synthesis and support informed decision-making, several AMSTAR appraisal initiatives have been undertaken across diverse healthcare domains [23–27]. However, despite the growing number of systematic reviews in resuscitation education, there has been no comprehensive assessment of their methodological quality, nor any systematic investigation into the factors influencing their rigor and reliability.

Accordingly, this review aimed to map the existing landscape of systematic reviews on resuscitation training interventions for healthcare professionals and to evaluate their methodological quality using standardized appraisal criteria. The findings are expected to inform best practices for conducting and reporting evidence syntheses in resuscitation education and to guide the development of higher-quality systematic reviews in the broader field of emergency and critical care training.

## Methods

This protocol was conducted in accordance with the *Preferred Reporting Items for Systematic Review and Meta-Analysis Protocols (PRISMA-P)* statement [15]. It has also been registered to the International Prospective Register of Systematic Reviews (PROSPERO) prior to the initiation of any data collection procedures, under the registration number CRD420251151633.

Ethical approval was not required for this study as it is a systematic review and meta-analysis of previously published research. All data analyzed were obtained from publicly available sources and contained no individual patient-identifiable information.

The systematic review was initiated in August 2025, with final completion anticipated in December 2026. Key methodological milestones include record screening (title/abstract and full-text review) slated for conclusion by March 31, 2026; data extraction targeted for completion by October 30, 2026; and the preparation of the final analysis and results manuscript expected by December 2026.

### Review questions

1. What is the scope and distribution of published systematic reviews on resuscitation training interventions?
2. What is the methodological quality of the systematic reviews?
3. What factors are associated with higher or lower methodological quality?
4. What methodological gaps in systematic reviews on resuscitation training need to be addressed to improve rigor and inform future training development?

### Studies criteria

Table 1 shows the full definition of our PICOST criteria. All published systematic reviews that evaluated resuscitation training interventions for healthcare professionals (physicians, nurses, paramedics, and other clinical personnel) and students will be included in this review. All types of training across different populations (adult, pediatric, neonates), including basic, advanced, or specialized resuscitation training (e.g., BLS, ACLS, PALS, NRP), were eligible, regardless of training format (e.g., face-to-face, simulation-based, e-learning, blended learning). All reviews focused on the evaluation of training with a specific procedure, and training for lay rescuers was excluded.

**Table 1:** Definition of eligibility criteria for article selection process.

Descriptor	Inclusion criteria	Exclusion criteria
Population	Health care professionals including medical staff, nurses, midwives, other allied healthcare workers, and healthcare-related students.	Lay people
Intervention	Any variant of resuscitation training instructional designs	Training focus on single procedure such as ventilation, intubation, AED only.
Control	All types of control/comparators.	There will be no restriction of study comparator
Outcome	Technical and non-technical performance, knowledge, CPR metrics,	None
Study design	Meta-analysis, systematic review, scoping review. Non-peer reviewed scholar articles such as thesis and dissertation will be part of our interest.	Less-methodological review such article review, integrative review, narrative review. Simulation trial studies including randomized trial, quasi experiment, pre-post test simulation trial. Abstract, and conference proceeding will be exempt from our review.
Time frame	Course conclusion and follow up	

Note: AED: Automated External Defibrillation; CPR: Cardiopulmonary Resuscitation.

This review will include systematic reviews of various experimental studies (RCTs, quasi-experiments, pre-post studies, observational studies, or qualitative studies) with or without meta-analysis, and without restriction on the year of publication or language. Additionally, original studies will be excluded, including RCTs, quasi-experimental studies, cross-sectional studies, and cohort studies, as well as proceedings papers.

### Search strategy

The lead researcher (DE) will develop, test, and refine the search strategy in collaboration with co-authors who have expertise in resuscitation training and evidence synthesis. The headings and keywords will be controlled based on the study’s aim, target condition (resuscitation), intervention type (training or educational programs), and study design (systematic reviews). Preliminary searches will be conducted to determine the initial search terms.

A comprehensive literature search will be conducted in the following databases: CENTRAL, Embase, MEDLINE, ProQuest, Scopus, Web of Science, CINAHL, and ERIC. Some repositories of reviews are also utilized in the searching process, such as: Cochrane database of Systematic Reviews, KSR Evidence, Epistemonikos, JBI Database of Systematic Reviews and Implementation Reports, and DARE [28]. Sample of search strategy can be found in S1 Table.

The review team will conduct additional hand searches in relevant databases and field-specific journals, such as Resuscitation, Clinical Simulation in Nursing, Simulation in Healthcare, and Resuscitation Plus, to enhance the finding of relevant records [29,30]. The search filter proposed by the National Library of Medicine will be used to identify systematic reviews [31]. The final search will be carried out using backward and forward citation tracing of the included articles [30]. All search results will be saved in the Rayyan web-based tool [32]. The full syntax is available in supported file.

### Study records

#### Selection process.

The titles and abstracts identified from the search process will be independently assessed for eligibility by two reviewers. They will assess the relevance of papers to the inclusion criteria. Studies that do not meet the criteria will be excluded at this stage. When the eligibility of a study cannot be determined solely by considering its abstract, the record will proceed to full-text screening.

The full texts of candidate eligible studies will subsequently be obtained and assessed independently by the two reviewers using the PICOST (Population, Intervention, Control, Outcome, Study design, Timeframe) framework [33]. During this stage, any article that does not meet the inclusion criteria will not be included with reasons recorded systematically (e.g., incorrect population, lack of training intervention, or not a systematic review). If any disagreement occurs between two reviewers, the principal investigator will be involved to reach a consensus.

**Fig 1:**
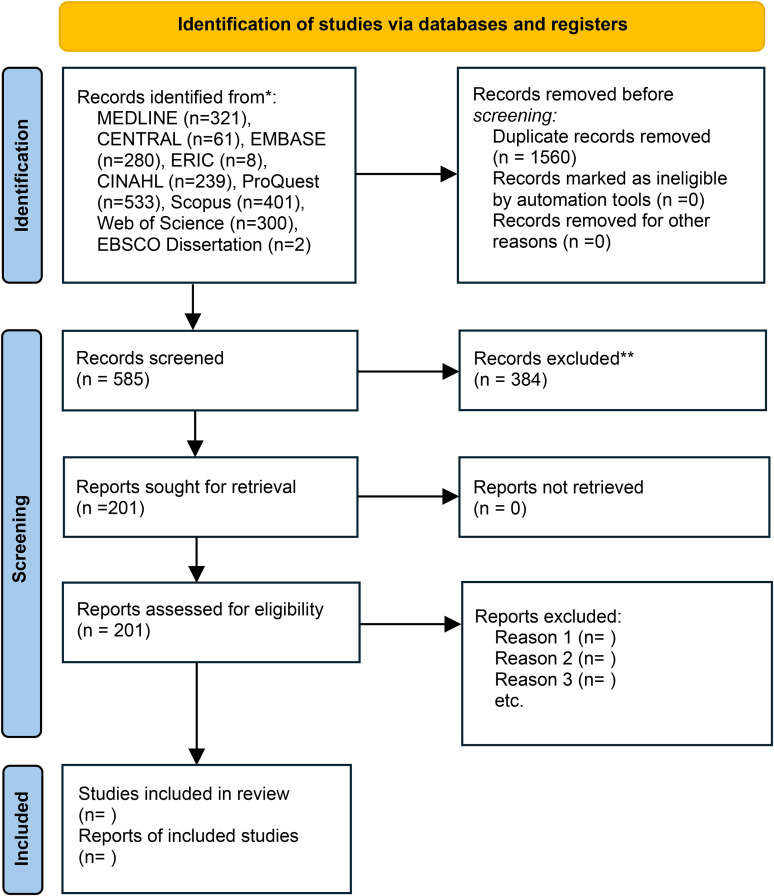
Study selection process.

A PRISMA flow diagram will be implemented to report all the selection process (Fig 1) [34]. This documentation will include the total number of records identified, the number after duplicate removal, the number screened, the number of full-text articles assessed, the reasons for exclusion, and the final number of studies included in the review. The data extraction and quality appraisal will be conducted only for studies that fully meet the predefined eligibility criteria, as determined through a full-text review process.

#### Data (items, management, and extraction).

Generally, information extraction will be divided into two types. First, information regarding publication, such as the first author, paper title, publication year, and database, will be summarized and presented in a table in Excel (v16.01, 2020). Second, the characteristics of the study will be outlined, including the study field, study design, population, and instructional design. The potential issue related to methodological quality will be discussed in a separate section. Gaps in the existing evidence synthesis will be identified and discussed. Two reviewers will conduct this process, and a third reviewer will play a role in reaching consensus when any discrepancies emerge. Details of the extraction items are shown in Table 2. The data supporting the findings of this study are derived from publicly available primary research articles cited in the reference list. The specific datasets extracted and analyzed during the current study are available from the corresponding author upon reasonable request. Such data will be accessible for public upon the publication of the systematic review.

**Table 2:** Sample of data extraction details.

Citation	Type of review	Topic	Objective	Population	Intervention and comparator	Study design	Result synthesis method	Number of authors	Country	Impact factor
										
										
										
										
										
										
										

#### The AMSTAR 2 items.

The methodological quality of the included systematic reviews will be assessed using the AMSTAR 2 instrument. As this review intends to map the methodological quality rather than the quality of evidence, therefore, instrument to analyze the rating evidence such as, GRADE (Grading of Recommendation, Assessment, Development, and Evaluation) will be not used in this study [28]. The AMSTAR 2 is a reliable and valid instrument for assessing the methodological quality of systematic reviews that involve both randomized and non-randomized healthcare intervention studies [20,35]. This tool requires 16 items that address essential methodological areas, including (1) evaluation of research question using the PICO (population, intervention, control, outcome) framework, (2) predetermined methods, (3) clear inclusion criteria, (4) exhaustive literature search, (5) duplicate study selection, (6) duplicate data extraction, (7) reported excluded studies, (8) detailed account of included studies, (9) risk of bias (RoB) assessment, (10) disclosed funding sources, (11) appropriate statistical methods for evidence synthesis, (12) quantitative evaluation of RoB in the main findings, (13) discussion of RoB at study level, (14) explained result heterogeneity, (15) examined publication bias, and (16) reported conflicts of interest. Detailed information for these items is presented in Table 3.

**Table 3:** Sixteen items in AMSTAR 2.

Item	Checklist items	Choice
1	Did the research questions and inclusion criteria for the review include the components of PICO?	Y/ N
2^#^	Did the report of the review contain an explicit statement that the review methods were established prior to the conduct of the review and did the report justify any significant deviations from the protocol?	Y/ PY/ N
3	Did the review authors explain their selection of the study designs for inclusion in the review?	Y/ N
4^#^	Did the review authors use a comprehensive literature search strategy?	Y/ PY/ N
5	Did the review authors perform study selection in duplicate?	Y/ N
6	Did the review authors perform data extraction in duplicate?	Y/ N
7^#^	Did the review authors provide a list of excluded studies and justify the exclusions?	Y/ PY/ N
8	Did the review authors describe the included studies in adequate detail?	Y/ PY/ N
9^#^	Did the review authors use a satisfactory technique for assessing the risk of bias (ROB) in individual studies that were included in the review?	Y/ PY/ N
10	Did the review authors report on the sources of funding for the studies included in the review?	Y/ N
11^#^	If meta-analysis was performed did the review authors use appropriate methods for statistical combination of results?	Y/ N/ N-MA
12	If meta-analysis was performed, did the review authors assess the potential impact of ROB in individual studies on the results of the meta-analysis or other evidence synthesis?	Y/ N/ N-MA
13^#^	Did the review authors account for ROB in individual studies when interpreting/discussing the results of the review?	Y/ N
14	Did the review authors provide a satisfactory explanation for, and discussion of, any heterogeneity observed in the results of the review?	Y/ N
15^#^	If they performed quantitative synthesis did the review authors carry out an adequate investigation of publication bias (small study bias) and discuss its likely impact on the results of the review?	Y/ N/ N-MA
16	Did the review authors report any potential sources of conflict of interest, including any funding they received for conducting the review?	Y/ N

Note: PICO: Population, Intervention, Control, and Outcome; #: Critical domain, Y: Yes; N: No; N-MA: No Meta-analysis conducted.

#### Methodological quality appraisal.

This assessment will be conducted independently by two reviewers, who will use AMSTAR 2 to appraise all included reviews. A pilot appraisal will be conducted on a subset of included studies before the comprehensive methodological appraisal, to ensure consistency and agreement between reviewers. Inter-rater agreement will be counted and each reviewer should pass an agreement score of more than 80% to continue the process. Each item of AMSTAR will be rated as “Yes,” “Partial Yes,” or “No” (item number 2, 4, 7, 8 and), “Yes” and “No” (item number 1, 3, 5, 6, 9, 10, 13, 14 and 16), and “Yes”, “No” and “No Meta-Analysis” (Item number 11, 12 and 14). The consistency of assessment will be assured by conducting calibration before the appraisal process. The calibration was conducted by jointly rating a small subset of studies and discussing the interpretation of each item until consensus was achieved. If any discrepancies emerge during the assessment process, they will first be resolved through discussion. If the discrepancy persists, the principal investigator will arbitrate to reach a final judgment.

The methodological quality of each systematic review will be summarized after all items have been checked for each paper. This step will be carried out by following the AMSTAR 2 overall confidence rating, which classifies reviews as high, moderate, low, or critically low. This global rating is determined by considering the presence and severity of critical and non-critical weaknesses across the items [20]. The range for the global rating is detailed in Table 4.

**Table 4:** Global Rating and Corresponding Range for AMSTAR 2.

Rate	Number of non-critical weakness	Number of critical flaw
High confidence	≤1	0
Moderate confidence	>1	0
Low confidence	Any	1
Critically low confidence	Any	>1

We will summarize the methodological quality of included systematic reviews using descriptive statistics. The frequency of responses for each AMSTAR 2 item, as well as the answers to all AMSTAR 2 items and their overall quality rating for each review, will be detailed in Table 5. This presentation will enable a clear assessment of evidence strengths and highlight potential methodological limitations.

**Table 5:** Evaluation of the methodological quality of reviews (AMSTAR-2).

No	Studies																	Overall judgement
		1	2	3	4	5	6	7	8	9	10	11	12	13	14	15	16	
																		
																		
																		
																		

Note: The overall quality will be categorized as critically low (CL), low (L), medium (M), and high (H).

### Data analysis and synthesis

The focus of this review is to map methodological aspects rather than to pool the intervention effect size of our included studies. Therefore, the findings will be reported in accordance with the synthesis without meta-analysis (SWiM) framework [36]. Previously, a matrix will be developed to identify overlapping primary studies, and the corrected covered area (CCA) index will be calculated across included systematic reviews [28]. CCA level of <5% indicates a mild overlap, 6–10% shows moderate overlap, while 11–15% and >15% are considered high and very high overlaps respectively [37]. Furthermore, graphical representation of overlap for overviews (GROOVE) will be utilized to present a graphical description of the overlapping studies to ensure transparency and accuracy [38]. A priori overlapping decision rule has been set: if the overlap is slight to moderate (CCA ≤ 10%), all reviews will be retained; if overlap is high or very high (CCA > 10%), we will prioritize and retain the most comprehensive and highest-quality systematic reviews based on methodological quality and recency. In case of sufficient data, this study will conduct regression analyses of interest a-priori to the review:

#### Type of review.

This study will identify all types of reviews, including systematic reviews with and without meta-analysis. This variation can provide the necessary variance in methodology, which is crucial for achieving the goal of this study. A scoping review will be considered if the study aims focus on assessing the efficacy of training.

#### Population: Healthcare staff, students, or both.

The population of interest will include healthcare staff (e.g., nurses, physicians, paramedics) and healthcare students (e.g., nursing and medical students). Studies involving mixed populations will also be included to capture the vast simulation applications across varying levels of clinical experience.

#### Number of simulation trials.

This study will inform the number of simulation trials included in this review. It is deemed important to highlight this information because simulation is a core educational method for resuscitation training. By doing so, the extent of evidence can be quantified, and the research gap can be identified.

#### Formal review of resuscitation bodies.

This study aims to identify the involvement of resuscitation organizations (e.g., ILCOR, AHA, ERC) in the recognition of included studies. Studies retrieved from formal reviews published by these organizations may offer high-level consensus and standardized practices.

#### Resuscitation topics.

In terms of topic, this study will categorize the included studies as Basic Life Support (BLS), Advanced Cardiac Life Support (ACLS), Pediatric Advanced Life Support (PALS), Neonatal Resuscitation Program (NRP), and Neonatal Life Support (NLS), as well as their combinations. This classification aims to identify the level of training most frequently addressed by simulation-based education.

- Number of authors

The number of authors will be documented from all included studies. This approach is necessary to identify the collaborative scope of each research project. Involving more authors may indicate better rigor.

- Country/region of the corresponding author’s affiliation

This study will map the distribution of authors worldwide based on the included studies. This information needs to be recorded to examine global research contributions, especially in the adoption of simulation for resuscitation training.

- Journal impact factor (by Clarivate)

The impact factor of the journal will be extracted from each included study. This metric is important for providing information regarding the reputation of outlets that publish simulation-based resuscitation studies.

- SSCI journal

It is also noted whether the journal in which the studies were included was indexed in the Social Science Citation Index (SSCI). This data will provide information regarding the quality of academic journals, particularly in educational and behavioral aspects of healthcare training.

- Open or close access journal

It will be necessary to recognize the type of journal based on its accessibility, such as open or closed access. This information will describe the accessibility of research findings to practitioners, educators, and the broader public.

- Sponsorship of individual studies

To evaluate potential conflicts of interest or bias, this study will record the presence or absence of funding or sponsorship for each included study. This sponsorship includes financial support from institutions, government bodies, or any grant for conducting research.

- Last search to first submission (month/year)

The duration between the last date of literature search and the first manuscript submission will be estimated for each included study. This number will inform regarding the time spent on the synthesis and reporting of data.

- Last search to publish (month/year)

This study will also report the time interval from the last literature search to the publication date. It is essential to evaluate the effectiveness of the peer-review and publication process.

### Statistical analysis

Regression analysis will be employed to investigate the impact of both categorical and continuous factors on the overall methodological quality of systematic reviews, as evaluated by the AMSTAR 2 tool. Categorical factors of interest will include review type (systematic review, meta-analysis), population (healthcare professionals, students, or both), resuscitation topic (e.g., BLS, ACLS, PALS, NRP), journal type (open access vs. closed access), journal indexing (SSCI vs. non-SSCI), and sponsorship status (funded vs. non-funded). Continuous factors will include the number of authors, journal impact factor, and time intervals (e.g., from last search to submission, from last search to publication). Associations will be tested using univariate regression, followed by multivariate models to account for potential confounders. Model assumptions will be assessed prior to interpretation. Results will be presented as regression coefficients (β) with 95% confidence intervals (CIs) and p-values.

All statistical analyses will be performed using Microsoft Excel 2019 for descriptive data handling and SPSS version 26.0 (IBM Corp., Armonk, NY, USA) for inferential analyses. Statistical significance will be set at 2-sided p < 0.05. The completeness of our protocol is reported in S2 Table.

## Results

The comprehensive processes of literature search, screening of studies, and extraction of relevant data commenced in August 2025, following the established protocol. The anticipated timeline for the completion of data analysis and synthesis is by February 2026.

## Discussion

Our study aims to deliver an in-depth and comprehensive synthesis of the methodological quality observed in published systematic reviews that focus on resuscitation training. By systematically evaluating these reviews using the AMSTAR 2 tool, this study will critically appraise the rigor, transparency, and reliability of their methodologies. The analysis will thereby provide valuable insights for researchers, educators, and policymakers by mapping the methodological landscape of evidence syntheses in resuscitation training. Ultimately, this study will support the advancement of higher standards in systematic review conduct and reporting within this vital area of healthcare education.

### Strengths

There have been numerous systematic reviews on resuscitation training, primarily focusing on evaluating the effectiveness of specific training strategies, such as the impact of simulation-based learning, debriefing methods, or digital platforms on knowledge and skill acquisition. To our present search, no comprehensive review of reviews has been conducted that systematically evaluates the methodological quality of systematic reviews themselves. Hence, the main strength of our review lies in its unique focus on appraising how well these reviews were conducted, rather than testing the effectiveness of a particular intervention. This review provides a transparent assessment of the robustness of reviews currently circulating in the literature, utilizing the AMSTAR 2 tools. Thus, the power and reliability of evidence across different types of resuscitation training interventions can be compared, which is imperative for both researchers and policymakers in advancing methodological rigor for better practice in resuscitation.

### Limitations

We must acknowledge that several limitations need to be anticipated. First, although no language restrictions are planned, this approach may result in subtle bias due to the translation process. Second, the reliability of the appraisal process may depend on how closely the published review complies with the standard of reporting, which can vary considerably. Lastly, given the heterogeneity of trainee populations including different age categories, training methods, and outcome measures across systematic reviews, this may challenge the finding synthesis consistently and constrain the generalizability of the findings.

## Conclusion

This systematic review of reviews will systematically map the distribution of published evidence syntheses on resuscitation training. This systematic review will also critically evaluate methodological quality, reporting factors associated with rigor and reliability, as well as methodological gaps to address in future studies. The findings are expected to culminate in recommendations that will serve as a fundamental foundation for guiding researchers in producing higher-quality systematic reviews and developing more effective methodological approaches. Additionally, this systematic review will guide educators and policymakers in designing effective resuscitation training programs, which are deemed essential to improving clinical outcomes in the practice of resuscitation worldwide.

## Supporting information

S1 Table This is the table of search results. (DOCX)

S2 Table This is the table of PRISMA checklist. (DOCX)
