# Supplementary material for: Mapping and appraising evidence syntheses of resuscitation training for healthcare professionals: Protocol for a systematic review of reviews
Source: PLoS One. 2026 Jul 13;21(7):e0349693. doi: 10.1371/journal.pone.0349693 (PMC13362104; doi:10.1371/journal.pone.0349693)
Supplement: S1 Table — (DOCX) [file pone.0349693.s001.docx]

**S1 Table**

1. CENTRAL

| ID | Search | Hits |
| --- | --- | --- |
| #1 | ("Resuscitation" OR "Life Support Care" OR "life support" OR "Cardiopulmonary Resuscitation" OR "Advanced Cardiac Life Support" OR "advanced trauma life support" OR "advanced trauma life support" OR "advanced life support" OR "basic life support" OR "advanced cardiac life" OR "emergency" OR "basic life" OR "rapid response" OR “chest compression” OR “helping babies breathe” OR “Helping Baby Breathe” OR HBB OR “First aid” OR “neonatal resuscitation” OR BLS OR ACLS OR NRP):ti AND (training OR education OR re-train OR refresher OR re-fresher OR teach* OR simulation OR simulations OR simulated OR simulat OR learning OR skill OR skills OR “problem-based” OR learning):ti (Word variations have been searched) | 2270 |
| #2 | (Review OR Reviews OR Scoping Review OR Systematic Review OR Meta-analysis OR Meta analysis OR "consensus conference" OR "critical review" OR "critical synthesis" OR "evidence synthesis" OR "integrative review" OR "integrative synthesis" OR "literature review" OR "meta-analysis" OR "meta-synthesis" OR "metastudy" OR "narrative review" OR "protocols" OR "qualitative review" OR "rapid review" OR "realist review" OR "review" OR "scoping" OR "systematic" OR "systematic Cochrane review" OR "systematic critical review" OR "systematic evidence review" OR "systematic integrative review" OR "systematic literature review" OR "systematic mapping review" OR "systematic meta-review" OR "systematic mixed studies review" OR "systematic narrative review" OR "systematic qualitative review" OR "systematic quantitative review" OR "systematic review" OR "systematic scoping review" OR "systematic search and review" OR "umbrella review"):ti (Word variations have been searched) | 55190 |
| #3 | #1 AND #2 | 61 |

1. CINAHL Plus

| ID | Search | Hits |
| --- | --- | --- |
| S1 | TI ("Resuscitation" OR "Life Support Care" OR "life support" OR "Cardiopulmonary  Resuscitation" OR "Advanced Cardiac Life Support" OR "advanced trauma life support" OR "advanced life support" OR "basic life support" OR "advanced cardiac life" OR "emergency" OR "basic life" OR "rapid response" OR “chest compression” OR “helping babies breathe” OR “Helping Baby Breathe” OR HBB OR “First aid” OR “neonatal resuscitation” OR BLS OR ACLS OR NRP):ti AND (training OR education OR re-train OR refresher OR re-fresher OR teach* OR simulation OR simulations OR simulated OR simulat OR learning OR skill OR skills OR “problem-based” OR learning):ti (Word variations have been searched) | 6707 |
| S2 | TI Review OR Reviews OR Scoping Review OR Systematic Review OR Meta-analysis OR  Meta analysis OR "consensus conference" OR "critical review" OR "critical synthesis" OR "evidence synthesis" OR "integrative review" OR "integrative synthesis" OR "literature review" OR "meta-analysis" OR "meta-synthesis" OR "metastudy" OR "narrative review" OR "protocols" OR "qualitative review" OR "rapid review" OR "realist review" OR "review" OR "scoping" OR "systematic" OR "systematic Cochrane review" OR "systematic critical review" OR "systematic evidence review" OR "systematic integrative review" OR "systematic literature review" OR "systematic mapping review" OR "systematic meta-review" OR "systematic mixed studies review" OR "systematic narrative review" OR "systematic qualitative review" OR "systematic quantitative review" OR "systematic review" OR "systematic scoping review" OR "systematic search and review" OR "umbrella review"):ti (Word variations have been searched) | 661897 |
| S3 | S1 AND S2 | 239 |

1. EBSCO Dissertation

| ID | Search | Hits |
| --- | --- | --- |
| S1 | TI ("Resuscitation" OR "Life Support Care" OR "life support" OR "Cardiopulmonary  Resuscitation" OR "Advanced Cardiac Life Support" OR "advanced trauma life support" OR "advanced life support" OR "basic life support" OR "advanced cardiac life" OR "emergency" OR "basic life" OR "rapid response" OR “chest compression” OR “helping babies breathe” OR “Helping Baby Breathe” OR HBB OR “First aid” OR “neonatal resuscitation” OR BLS OR ACLS OR NRP):ti AND (training OR education OR re-train OR refresher OR re-fresher OR teach* OR simulation OR simulations OR simulated OR simulat OR learning OR skill OR skills OR “problem-based” OR learning):ti (Word variations have been searched) | 174 |
| S2 | TI Review OR Reviews OR Scoping Review OR Systematic Review OR Meta-analysis OR  Meta analysis OR "consensus conference" OR "critical review" OR "critical synthesis" OR "evidence synthesis" OR "integrative review" OR "integrative synthesis" OR "literature review" OR "meta-analysis" OR "meta-synthesis" OR "metastudy" OR "narrative review" OR "protocols" OR "qualitative review" OR "rapid review" OR "realist review" OR "review" OR "scoping" OR "systematic" OR "systematic Cochrane review" OR "systematic critical review" OR "systematic evidence review" OR "systematic integrative review" OR "systematic literature review" OR "systematic mapping review" OR "systematic meta-review" OR "systematic mixed studies review" OR "systematic narrative review" OR "systematic qualitative review" OR "systematic quantitative review" OR "systematic review" OR "systematic scoping review" OR "systematic search and review" OR "umbrella review"):ti (Word variations have been searched) | 6912 |
| S3 | S1 AND S2 | 2 |

1. ERIC via EBSCHO

| ID | Search | Hits |
| --- | --- | --- |
| S1 | TI ("Resuscitation" OR "Life Support Care" OR "life support" OR "Cardiopulmonary  Resuscitation" OR "Advanced Cardiac Life Support" OR "advanced trauma life support" OR "advanced life support" OR "basic life support" OR "advanced cardiac life" OR "emergency" OR "basic life" OR "rapid response" OR “chest compression” OR “helping babies breathe” OR “Helping Baby Breathe” OR HBB OR “First aid” OR “neonatal resuscitation” OR BLS OR ACLS OR NRP):ti AND (training OR education OR re-train OR refresher OR re-fresher OR teach* OR simulation OR simulations OR simulated OR simulat OR learning OR skill OR skills OR “problem-based” OR learning):ti (Word variations have been searched) | 484 |
| S2 | TI Review OR Reviews OR Scoping Review OR Systematic Review OR Meta-analysis OR  Meta analysis OR "consensus conference" OR "critical review" OR "critical synthesis" OR "evidence synthesis" OR "integrative review" OR "integrative synthesis" OR "literature review" OR "meta-analysis" OR "meta-synthesis" OR "metastudy" OR "narrative review" OR "protocols" OR "qualitative review" OR "rapid review" OR "realist review" OR "review" OR "scoping" OR "systematic" OR "systematic Cochrane review" OR "systematic critical review" OR "systematic evidence review" OR "systematic integrative review" OR "systematic literature review" OR "systematic mapping review" OR "systematic meta-review" OR "systematic mixed studies review" OR "systematic narrative review" OR "systematic qualitative review" OR "systematic quantitative review" OR "systematic review" OR "systematic scoping review" OR "systematic search and review" OR "umbrella review"):ti (Word variations have been searched) | 12011 |
| S3 | S1 AND S2 | 8 |

1. EMBASE

| No | Query Results | Results |
| --- | --- | --- |
| #1 | ('resuscitation':ti OR 'life support care':ti OR 'life support':ti OR 'cardiopulmonary resuscitation':ti OR 'advanced cardiac life support':ti OR 'advanced trauma life support':ti OR 'advanced life support':ti OR 'basic life support':ti OR 'advanced cardiac life':ti OR 'emergency':ti OR 'basic life':ti OR 'rapid response':ti OR 'chest compression':ti OR 'helping babies breathe':ti OR 'helping baby breathe':ti OR hbb:ti OR 'first aid':ti OR 'neonatal resuscitation':ti OR bls:ti OR acls:ti OR nrp:ti) AND (training:ti OR education:ti OR 're train':ti OR refresher:ti OR 're fresher':ti OR teach*:ti OR simulation:ti OR simulations:ti OR simulated:ti OR simulat:ti OR skill:ti OR skills:ti OR 'problem-based':ti OR learning:ti) AND ((review:ti OR reviews:ti OR 'scoping review':ti OR 'meta analysis':ti OR 'consensus conference':ti OR 'critical review':ti OR 'critical synthesis':ti OR 'evidence synthesis':ti OR 'integrative review':ti OR 'integrative synthesis':ti OR 'literature review':ti OR 'meta-analysis':ti OR 'meta-synthesis':ti OR 'metastudy':ti OR 'narrative review':ti OR 'protocols':ti OR 'qualitative review':ti OR 'rapid review':ti OR 'realist review':ti OR 'review':ti OR 'scoping':ti OR 'systematic':ti OR 'systematic cochrane review':ti OR 'systematic critical review':ti OR 'systematic evidence review':ti OR 'systematic integrative review':ti OR 'systematic literature review':ti OR 'systematic mapping review':ti OR 'systematic meta-review':ti OR 'systematic mixed studies review':ti OR 'systematic narrative review':ti OR 'systematic qualitative review':ti OR 'systematic quantitative review':ti OR 'systematic review':ti OR 'systematic scoping review':ti OR 'systematic search':ti) AND review:ti OR 'umbrella review':ti) | 280 |

1. PROQUEST

| ID | Search | Hits |
| --- | --- | --- |
| S1 | title("Resuscitation" OR "Life Support Care" OR "life support"OR "Cardiopulmonary Resuscitation" OR "Advanced Cardiac Life Support" OR "advanced trauma life support" OR "advanced trauma life  support" OR "advanced life support" OR "basic life support" OR "advanced cardiac life" OR "emergency" OR "basic life" OR "rapid response" OR “chest compression” OR “helping babies breathe” OR “Helping Baby Breathe” OR HBB OR “First aid” OR “neonatal resuscitation” OR BLS OR ACLS OR NRP) AND title(training OR education OR re-train OR refresher OR refresher OR teach* OR simulation OR simulations OR simulated OR simulat OR learning OR skill OR skills OR “problem-based” OR learning) | 15778 |
| S2 | title(Review OR Reviews OR Scoping Review OR Systematic Review OR Meta-analysis OR Meta analysis OR "consensus conference" OR "critical review" OR "critical synthesis" OR "evidence synthesis" OR "integrative review" OR "integrative synthesis" OR "literature review" OR "meta-  analysis" OR "meta-synthesis" OR "metastudy" OR "narrative review" OR "protocols" OR "qualitative review" OR "rapid review" OR "realist review" OR "review" OR "scoping" OR "systematic" OR "systematic Cochrane review" OR "systematic critical review" OR "systematic evidence review" OR "systematic integrative review" OR "systematic literature review" OR "systematic mapping review" OR "systematic meta-review" OR "systematic mixed studies review" OR "systematic narrative review" OR "systematic qualitative review" OR "systematic quantitative review" OR "systematic review" OR "systematic scoping review" OR "systematic search and review" OR "umbrella review") | 1698763 |
| S3 | S1 AND S2 | 533 |

1. MEDLINE via PUBMED

| Search number | Query | Results |
| --- | --- | --- |
| 5 | (("Resuscitation"[Title] OR "Life Support Care"[Title] OR "life support"[Title] OR "Cardiopulmonary Resuscitation"[Title] OR "Advanced Cardiac Life Support"[Title] OR "advanced trauma life support"[Title] OR "advanced trauma life support"[Title] OR "advanced life support"[Title] OR "basic life support"[Title] OR "advanced cardiac life"[Title] OR "emergency"[Title] OR "basic life"[Title] OR "rapid response"[Title] OR "chest compression"[Title] OR "helping babies breathe"[Title] OR "Helping Baby Breathe"[Title] OR HBB[Title] OR "First aid"[Title] OR "neonatal resuscitation"[Title] OR BLS[Title] OR ACLS[Title] OR NRP[Title]) AND (training[Title] OR education[Title] OR re-train[Title] OR refresher[Title] OR re-fresher[Title] OR teach*[Title] OR simulation[Title] OR simulations[Title] OR simulated[Title] OR simulat[Title] OR learning[Title] OR skill[Title] OR skills[Title] OR "problem-based"[Title] OR learning[Title])) AND (Review[Title] OR Reviews[Title] OR Scoping Review[Title] OR Systematic Review[Title] OR Meta-analysis[Title] OR Meta analysis[Title] OR "consensus conference"[Title] OR "critical review"[Title] OR "critical synthesis"[Title] OR "evidence synthesis"[Title] OR "integrative review"[Title] OR "integrative synthesis"[Title] OR "literature review"[Title] OR "meta-analysis"[Title] OR "meta-synthesis"[Title] OR "metastudy"[Title] OR "narrative review"[Title] OR "protocols"[Title] OR "qualitative review"[Title] OR "rapid review"[Title] OR "realist review"[Title] OR "review"[Title] OR "scoping"[Title] OR "systematic"[Title] OR "systematic Cochrane review"[Title] OR "systematic critical review"[Title] OR "systematic evidence review"[Title] OR "systematic integrative review"[Title] OR "systematic literature review"[Title] OR "systematic mapping review"[Title] OR "systematic meta-review"[Title] OR "systematic mixed studies review"[Title] OR "systematic narrative review"[Title] OR "systematic qualitative review"[Title] OR "systematic quantitative review"[Title] OR "systematic review"[Title] OR "systematic scoping review"[Title] OR "systematic search and review"[Title] OR "umbrella review"[Title]) | 321 |
| 4 | Review[Title] OR Reviews[Title] OR Scoping Review[Title] OR Systematic Review[Title] OR Meta-analysis[Title] OR Meta analysis[Title] OR "consensus conference"[Title] OR "critical review"[Title] OR "critical synthesis"[Title] OR "evidence synthesis"[Title] OR "integrative review"[Title] OR "integrative synthesis"[Title] OR "literature review"[Title] OR "meta-analysis"[Title] OR "meta-synthesis"[Title] OR "metastudy"[Title] OR "narrative review"[Title] OR "protocols"[Title] OR "qualitative review"[Title] OR "rapid review"[Title] OR "realist review"[Title] OR "review"[Title] OR "scoping"[Title] OR "systematic"[Title] OR "systematic Cochrane review"[Title] OR "systematic critical review"[Title] OR "systematic evidence review"[Title] OR "systematic integrative review"[Title] OR "systematic literature review"[Title] OR "systematic mapping review"[Title] OR "systematic meta-review"[Title] OR "systematic mixed studies review"[Title] OR "systematic narrative review"[Title] OR "systematic qualitative review"[Title] OR "systematic quantitative review"[Title] OR "systematic review"[Title] OR "systematic scoping review"[Title] OR "systematic search and review"[Title] OR "umbrella review"[Title] | 1005719 |
| 3 | ("Resuscitation"[Title] OR "Life Support Care"[Title] OR "life support"[Title] OR "Cardiopulmonary Resuscitation"[Title] OR "Advanced Cardiac Life Support"[Title] OR "advanced trauma life support"[Title] OR "advanced trauma life support"[Title] OR "advanced life support"[Title] OR "basic life support"[Title] OR "advanced cardiac life"[Title] OR "emergency"[Title] OR "basic life"[Title] OR "rapid response"[Title] OR "chest compression"[Title] OR "helping babies breathe"[Title] OR "Helping Baby Breathe"[Title] OR HBB[Title] OR "First aid"[Title] OR "neonatal resuscitation"[Title] OR BLS[Title] OR ACLS[Title] OR NRP[Title]) AND (training[Title] OR education[Title] OR re-train[Title] OR refresher[Title] OR re-fresher[Title] OR teach*[Title] OR simulation[Title] OR simulations[Title] OR simulated[Title] OR simulat[Title] OR learning[Title] OR skill[Title] OR skills[Title] OR "problem-based"[Title] OR learning[Title]) | 9401 |
| 2 | training[Title] OR education[Title] OR re-train[Title] OR refresher[Title] OR re-fresher[Title] OR teach*[Title] OR simulation[Title] OR simulations[Title] OR simulated[Title] OR simulat[Title] OR learning[Title] OR skill[Title] OR skills[Title] OR "problem-based"[Title] OR learning[Title] | 753442 |
| 1 | "Resuscitation"[Title] OR "Life Support Care"[Title] OR "life support"[Title] OR "Cardiopulmonary Resuscitation"[Title] OR "Advanced Cardiac Life Support"[Title] OR "advanced trauma life support"[Title] OR "advanced trauma life support"[Title] OR "advanced life support"[Title] OR "basic life support"[Title] OR "advanced cardiac life"[Title] OR "emergency"[Title] OR "basic life"[Title] OR "rapid response"[Title] OR "chest compression"[Title] OR "helping babies breathe"[Title] OR "Helping Baby Breathe"[Title] OR HBB[Title] OR "First aid"[Title] OR "neonatal resuscitation"[Title] OR BLS[Title] OR ACLS[Title] OR NRP[Title] | 165735 |

1. Scopus

| Search number | Query | Results |
| --- | --- | --- |
| 6 | ( ( TITLE ( "resuscitation" or "life support care" or "life support" or "cardiopulmonary resuscitation" or "advanced cardiac life support" or "advanced trauma life support" or"advanced trauma life support" or "advanced life support" or "basic life support" or "advanced cardiac life" or "emergency" or "basic life" or "rapid response" or "chest compression" or "helping babies breathe" or "helping baby breathe" or hbb or "first aid" or "neonatal resuscitation" or bls or acls or nrp ) ) and ( TITLE ( training or education or re-train or refresher or re-fresher or teach* or simulation or simulations or simulated or simulat or learning or skill or skills or "problem-based" or learning ) ) ) and ( TITLE ( review or reviews or scoping review or systematic review or meta-analysis or meta analysis or"consensus conference" or "critical review" or "critical synthesis" or "evidence synthesis" or "integrative  review" or "integrative synthesis" or "literature review" or "meta-analysis" or "meta-synthesis" or" metastudy" or "narrative review" or "protocols" or "qualitative review" or" rapid review" or" realist review" or "review" or "scoping" or"systematic" or "systematic cochrane review" or "systematic critical review" or "systematic evidence review" or "systematic integrative review" or "systematic literature review" or"systematic mapping review" or"systematic meta-review" or"systematic mixed studies review" or"systematic narrative review" or"systematic qualitative review" or"systematic quantitative review"  or"systematic review" or"systematic scoping review" or"systematic search and review" or"umbrella review" ) ) | 401 |
| 5 | TITLE ( review or reviews or scoping review or systematic review or meta-analysis or meta analysis or"consensus conference" or"critical review" or "critical synthesis" or"evidence synthesis" or"integrative review" or "integrative synthesis" or"literature review" or"meta-analysis" or"meta-synthesis" or"metastudy" or"narrative review" or"protocols" or"qualitative review" or"rapid review" or"realist review" or"review" or"scoping" or "systematic" or"systematic cochrane review" or"systematic critical review" or "systematic evidence review" or"systematic integrative review" or"systematic literature review" or"systematic mapping review" or"systematic meta-review" or"systematic mixed studies review" or"systematic narrative review" or "systematic qualitative review" or"systematic quantitative review" or "systematic review" or"systematic scoping review" or"systematic search and review" or"umbrella review" ) | 1464030 |
| 3 | ( TITLE ( "resuscitation" or"life support care" or"life support" or "cardiopulmonary resuscitation" or"advanced cardiac life support" or "advanced trauma life support" or"advanced trauma life support" or "advanced life support" or"basic life support" or"advanced cardiac life" or "emergency" or"basic life" or"rapid response" or"chest compression" or "helping babies breathe" or"helping baby breathe" or hbb or"first aid" or "neonatal resuscitation" or bls or acls or nrp ) ) and ( TITLE ( training or education or re-train or refresher or re-fresher or teach* or simulation or simulations or simulated or simulat or learning or skill or skills or"problem-based" or learning ) ) | 15792 |
| 2 | TITLE ( training or education or re-train or refresher or re-fresher or teach* or simulation or simulations or simulated or simulat or learning or skill or skills or "problem-based" or learning ) | 3410659 |
| 1 | TITLE ( "resuscitation" or"life support care" or"life support" or "cardiopulmonary resuscitation" or"advanced cardiac life support" or "advanced trauma life support" or"advanced trauma life support" or "advanced life support" or"basic life support" or"advanced cardiac life" or "emergency" or"basic life" or"rapid response" or"chest compression" or "helping babies breathe" or"helping baby breathe" or hbb or"first aid" or "neonatal resuscitation" or bls or acls or nrp ) | 255442 |

1. Web of science

| Search number | Query | Results |
| --- | --- | --- |
| 1 | "Resuscitation" OR "Life Support Care" OR "life support" OR "Cardiopulmonary Resuscitation" OR "Advanced Cardiac Life Support" OR "advanced trauma life support" OR "advanced trauma life support" OR "advanced life support" OR "basic life support" OR "advanced cardiac life" OR "emergency" OR "basic life" OR "rapid response" OR “chest compression” OR “helping babies breathe” OR “Helping Baby Breathe” OR HBB OR “First aid” OR “neonatal resuscitation” OR  BLS OR ACLS OR NRP (Title) | 169468 |
| 2 | training OR education OR re-train OR refresher OR re-fresher OR teach* OR simulation OR simulations OR simulated OR simulat OR learning OR skill OR skills OR “problem-based” OR learning (Title) | 1892795 |
| 3 | #1 AND #2 | 10205 |
| 4 | TI=(Review OR Reviews OR Scoping Review OR Systematic Review OR Meta-analysis OR Meta analysis OR "consensus conference" OR "critical review" OR "critical synthesis" OR "evidence synthesis" OR "integrative review" OR "integrative synthesis" OR "literature review" OR "meta-analysis" OR "meta-synthesis" OR "metastudy" OR "narrative review" OR "protocols" OR "qualitative review" OR "rapid review" OR "realist review" OR "review" OR "scoping" OR "systematic" OR "systematic Cochrane review" OR "systematic critical review" OR "systematic evidence review" OR "systematic integrative review" OR "systematic literature review" OR "systematic mapping review" OR "systematic meta-review" OR "systematic mixed studies review" OR "systematic narrative review" OR "systematic qualitative review" OR "systematic quantitative review" OR "systematic review" OR "systematic scoping review" OR "systematic search and review" OR "umbrella review") | 1234304 |
| 5 | #3 AND #4 | 300 |
