# Supplementary material for: Mapping and appraising evidence syntheses of resuscitation training for healthcare professionals: Protocol for a systematic review of reviews
Source: PLoS One. 2026 Jul 13;21(7):e0349693. doi: 10.1371/journal.pone.0349693 (PMC13362104; doi:10.1371/journal.pone.0349693)
Supplement: Attachment — Submitted filename: Response to rev_revDE1_260427.docx [file pone.0349693.s004.docx]

Dear Editor and Reviewers,

We deeply appreciate for the meticulous evaluation of our manuscript “Mapping and appraising evidence syntheses of resuscitation training for healthcare professionals: Protocol for a systematic review of reviews”. We greatly thank for the opportunity to revise the manuscript according to the constructive comments and feedback, which have helped us to enhance the clarity and methodological transparency of the manuscript.

Responding to the thorough evaluation, the manuscript had been revised according to the editor and both reviewers’ comments. Key revisions include: (1) Adding the global evidence of the survival rate of CPR; (2) Ensuring that the ethics statement is outlined in the method section; (3) Enhancing the data availability statement in the title page section; (4) Refining the methodological drawbacks in the protocol by expanding systematic review repositories in the literature search, adding overlapping primary studies identification and management as well as utilizing GROOVE, conducting a pilot quality appraisal, and including result synthesis method in the data extraction form; (5) Enhancing the availability of all data underlying the finding in the manuscript; (6) Incorporating the challenges to the analysis due to the heterogeneity in the limitation section; and (7) Listing all the individual authors and their affiliations of the LIFESAVER Consortium in the acknowledgement section.

We provide a detailed, point by point response to each comment in the following tables. We had also made revisions in the manuscript through yellow highlighted texts for convenience. Thank for your time and consideration. Hopefully, the updated manuscript is now suitable for publication. Should you have any inquiries, do not hesitate to contact us.

Best Regards,

WONG Cho Lee, PhD, FHKAN, APN

| No | Reviewer’s comments | Authors’ responses | Page No |
| --- | --- | --- | --- |
| **Introduction** |  |  |  |
| Reviewer 1 | Line 30 and introduction-The number of systematic reviews on resuscitation training has grown rapidly, offering valuable 32 evidence to guide healthcare decision-making- Please add global evidence of clear guidance on how much it saves lives. | Thank you for the important suggestion. We have added a sentence to show the global evidence of the survival rate of CPR.  “Evidence shows that cardiopulmonary resuscitations (CPR) increase global survival rates of out-of-hospital cardiac arrest (OHCA) from around 1% to 5% in the past 40 years [1]”. | Page 4 line 72-73 |
| **Methods** |  |  |  |
| Editor | Your ethics statement should only appear in the Methods section of your manuscript. If your ethics statement is written in any section besides the Methods, please move it to the Methods section and delete it from any other section. Please ensure that your ethics statement is included in your manuscript, as the ethics statement entered into the online submission form will not be published alongside your manuscript. | Thank you for the valuable suggestion. We have added and ensured that the ethical considerations are outlined within the method section.  “Ethical approval was not required for this study as it is a systematic review and meta-analysis of previously published research. All data analyzed were obtained from publicly available sources and contained no individual patient-identifiable information” | Page 17 line 129-131 |
|  | This is an umbrella review and the single most consequential methodological challenge unique to this design is the management of overlapping primary studies across included systematic reviews. When the same underlying trials appear in multiple SRs, double-counting inflates the apparent evidence base and distorts synthesis. This issue is well established in the methodological literature (Abdellatif et al., Trop Med Health, 2025; <https://doi.org/10.1186/s41182-025-00764-y>).  The current protocol contains no plan to address this. Specifically, it lacks:  1. A primary-study overlap matrix identifying which studies appear across which SRs.  2. Calculation of the Corrected Covered Area (CCA) index, the standard metric for quantifying overlap (slight <5%, moderate 6–10%, high 11–15%, very high >15%). | We thank the editor for the constructive feedback. We fully agree that managing overlap of primary studies is important in umbrella reviews. In response, we have revised our protocol to explicitly address this concern.  “Previously, a matrix will be developed to identify overlapping primary studies and the corrected covered area (CCA) index will be calculated across included systematic reviews [28]. CCA level of <5% indicates a mild overlap, 6-10% shows moderate overlap, while 11-15% and >15% are considered high and very high overlaps respectively [37].” | Page 17 line 288-292 |
|  | 3. Use a specifically developed and appropriately referenced CCA calculation tool (i.e., GROOVE - Pérez-Bracchiglione et al., Res Synth Methods, 2022; doi:10.1002/jrsm.1557). | Thank you for the suggestion. We do agree to use GROOVE for graphical description of the overlapping studies.  “Furthermore, graphical representation of overlap for overviews (GROOVE) will be utilized to present a graphical description of the overlapping studies to ensure transparency and accuracy [38].” | Page 17 line 292-294 |
|  | 4. A pre-specified decision rule for managing overlap contingent on the CCA level found. | We thank the editor for this thorough comment. We have added the strategy to manage the overlapping studies based on CCA level. The addition has been incorporated into the Methods section of the revised protocol.  “A priori overlapping decision rule has been set: if the overlap is slight to moderate (CCA ≤10%), all reviews will be retained; if overlap is high or very high (CCA >10%), we will prioritize and retain the most comprehensive and highest-quality systematic reviews based on methodological quality and recency.” | Page 17 line 294-297 |
|  | Search scope: Dedicated SR repositories (Cochrane Library, Epistemonikos, JBI Database, DARE) are absent from the search strategy. These are specifically recommended for umbrella reviews (Abdellatif et al., 2025) and their omission risks missing high-quality SRs not indexed in standard bibliographic databases. | Thank you for the valuable suggestion. Some SR repositories have been added in the searching method:  “Some repositories of reviews are also utilized in the searching process, such as: Cochrane database of Systematic Reviews, KSR Evidence, Epistemonikos, JBI Database of Systematic Reviews and Implementation Reports, and DARE” [28]. | Page 9 line 174-177 |
|  | GRADE: If GRADE is intentionally excluded because this review focuses on methodological mapping rather than intervention synthesis, this rationale must be stated explicitly in the Methods. | We thank the editor for the esteemed comments. We have added the statement to exclude GRADE in the Methods.  “As this review intends to map the methodological quality rather than the quality of evidence, therefore, instrument to analyze the rating evidence such as, GRADE (Grading of Recommendation, Assessment, Development, and Evaluation) will not be used in this study [28]” | Page 12 line 231-234 |
|  | Data extraction: The extraction form (Table 2) does not capture the results synthesis method of each included SR. Given the review's methodological focus, this is a relevant variable. | Thank you for this valuable suggestion. We agree that the method of results synthesis used in each included systematic review is an important variable, particularly given the methodological focus of this umbrella review.  In response, we have revised the data extraction form (Table 2) to include a specific item capturing the result synthesis method of each review | Page 11 line 224 |
| Reviewer 1 | Have the authors described where all data underlying the findings will be made available when the study is complete?  The [PLOS Data policy](https://track.editorialmanager.com/CL0/https:%2F%2Fjournals.plos.org%2Fplosone%2Fs%2Fmaterials-and-software-sharing/1/010f019d08e97a19-b5cdd476-b728-4918-8cbd-e83d23c98bfe-000000/vKKcpIIfhsC99-QGo-GylJA7LPu_HnGlHk9yRL1JvPc=255) requires authors to make all data underlying the findings described in their manuscript fully available without restriction, with rare exception, at the time of publication. The data should be provided as part of the manuscript or its supporting information, or deposited to a public repository. For example, in addition to summary statistics, the data points behind means, medians and variance measures should be available. If there are restrictions on publicly sharing data—e.g. participant privacy or use of data from a third party—those must be specified. | Thank you for this clarification. Based on the PLOS Data Availability policy, all data underlying the findings of this umbrella review will be fully available without restriction. The extracted data and others will be provided as supplementary files accompanying the manuscript.  “All data underlying the findings will be ensured available within the manuscript or supplementary files accompanying the manuscript.” | Page 10 line 220-221 |
|  | Line 107- include PROSPERO registration number | We thank the reviewer for the suggestion. We have added the PROSPERO registration number.  “It has also been registered to the International Prospective Register of Systematic Reviews (PROSPERO) prior to the initiation of any data collection procedures, under the registration number CRD420251151633”. | Page 6 line 126-128 |
|  | What is the scope and distribution of published systematic reviews on resuscitation 115 training interventions? Are you considering adult and child neonatal or all?? | Thank you for this clarification. This study includes systematic reviews on all types of resuscitation training interventions, including basic life support (BLS), advanced cardiovascular life support (ACLS), pediatric advanced life support (PALS), and neonatal resuscitation training (NRP). This clarification has been incorporated into the Methods section.  “All published systematic reviews that evaluated resuscitation training interventions for healthcare professionals (physicians, nurses, paramedics, and other clinical personnel) and students will be included in this review. All types of training across different populations (adult, pediatric, neonates), including basic, advanced, or specialized resuscitation training (e.g., BLS, ACLS, PALS, NRP), were eligible, regardless of training format (e.g., face-to-face, simulation-based, e-learning, blended learning).” | Page 7 line 146-151 |
|  | Pls state Methodological quality appraisal-will you plan for do piloting?? | Thank you for the thorough comment. We have added a pilot quality appraisal process conducted on a subset of included studies to ensure consistency and agreement between reviewers.  “A pilot appraisal will be conducted on a subset of included studies before the comprehensive methodological appraisal, to ensure consistency and agreement between reviewers. Inter-rater agreement will be counted and each reviewer should pass an agreement score of more than 80% to continue the process.” | Page 14 line 253-256 |
| Reviewer 2 | This is a Protocol for review of reviews . Clear and sound design. Looking forward for the actual review - study | Thank you for the compliment. The protocol will be developed to result in comprehensive and quality findings. |  |
| **Limitation** |  |  |  |
| Reviewer 1 | add more limitations different SR on age categories, different methods difficult to analysis | Thank you for the feedback. The limitation has incorporated in the manuscript:  “Lastly, given the heterogeneity of trainee populations including different age categories, training methods, and outcome measures across systematic reviews, this may challenge the finding synthesis consistently and constrain the generalizability of the findings.” | Page 22 line 410-412 |
|  |  |  |  |

Additional documents

| No | Documents | Authors’ responses |
| --- | --- | --- |
| 1 | your manuscript meets PLOS ONE's style requirements, including those for file naming | Thank you for the suggestion. We have ensured that the manuscript meets the PLOS ONE’s style requirements including for the file naming |
| 2 | a complete Data Availability Statement (please kindly revise your statement to explain your reasoning and we will seek the editor's input on an exemption) | Thank you for this suggestion. We have revised the Data Availability Statement to clarify our reasoning. As this is a systematic review and network meta-analysis, the study does not report primary data. All data were extracted from previously published clinical trials. Because the raw data belongs to the original trial investigators and is already in the public domain via their respective publications, we believe a formal data repository deposit is not applicable. However, we have archived our extraction tables and are prepared to share our synthesized data sheets with any researcher upon request to ensure transparency and reproducibility.  We have revised this statement in the title page. |
| 3 | Consortium “LIVESAFER (Leading Innovations for Emergency Support and Vital Enhancements in Resuscitation) Study Network. In addition to naming the author group, please list the individual authors and affiliations within this group in the acknowledgments section of your manuscript. Please also indicate clearly a lead author for this group along with a contact email address. | We thank the editor for the valuable advice. We have listed all individual authors and their affiliations of the LIVESAFERS Consortium along with the consortium leader in the acknowledgement section. This revision is placed in page 22-23, line 429-434. |
| 4 | Your ethics statement should only appear in the Methods section of your manuscript. | Thank you for the valuable suggestion. We have added and ensured that the ethical considerations are outlined within the method section |
