# Supplementary material for: Mapping and appraising evidence syntheses of resuscitation training for healthcare professionals: Protocol for a systematic review of reviews
Source: PLoS One. 2026 Jul 13;21(7):e0349693. doi: 10.1371/journal.pone.0349693 (PMC13362104; doi:10.1371/journal.pone.0349693)
Supplement: Peer review history

# Peer review: Decision Letter 0

**DOI:** 10.1371/journal.pone.0349693.r001

**Authors:** Athanasios Pantelis

19 Mar 2026

PONE-D-25-60471

Mapping and Appraising Evidence Syntheses of Resuscitation Training for Healthcare Professionals: Protocol for a Systematic Review of Reviews

PLOS One

Dear Dr.  Wong,

Thank you for submitting your manuscript to PLOS ONE. After careful consideration, we feel that it has merit but does not fully meet PLOS ONE’s publication criteria as it currently stands. Therefore, we invite you to submit a revised version of the manuscript that addresses the points raised during the review process.

==============================

**ACADEMIC EDITOR: No CCA analysis performed. Please see my comments below.**

==============================

Please submit your revised manuscript by May 03 2026 11:59PM. If you will need more time than this to complete your revisions, please reply to this message or contact the journal office at plosone@plos.org. When you're ready to submit your revision, log on to https://www.editorialmanager.com/pone/ and select the 'Submissions Needing Revision' folder to locate your manuscript file.

Please include the following items when submitting your revised manuscript:

- A letter that responds to each point raised by the academic editor and reviewer(s). You should upload this letter as a separate file labeled 'Response to Reviewers'.
- A marked-up copy of your manuscript that highlights changes made to the original version. You should upload this as a separate file labeled 'Revised Manuscript with Track Changes'.
- An unmarked version of your revised paper without tracked changes. You should upload this as a separate file labeled 'Manuscript'.

If you would like to make changes to your financial disclosure, please include your updated statement in your cover letter. Guidelines for resubmitting your figure files are available below the reviewer comments at the end of this letter.

If applicable, we recommend that you deposit your laboratory protocols in protocols.io to enhance the reproducibility of your results. Protocols.io assigns your protocol its own identifier (DOI) so that it can be cited independently in the future. For instructions see: https://journals.plos.org/plosone/s/submission-guidelines#loc-laboratory-protocols. Additionally, PLOS ONE offers an option for publishing peer-reviewed Lab Protocol articles, which describe protocols hosted on protocols.io. Read more information on sharing protocols at https://plos.org/protocols?utm_medium=editorial-email&utm_source=authorletters&utm_campaign=protocols.

We look forward to receiving your revised manuscript.

Kind regards,

Athanasios G. Pantelis

Academic Editor

PLOS One

Journal Requirements:

When submitting your revision, we need you to address these additional requirements.

1.Please ensure that your manuscript meets PLOS ONE's style requirements, including those for file naming. The PLOS ONE style templates can be found at

https://journals.plos.org/plosone/s/file?id=wjVg/PLOSOne_formatting_sample_main_body.pdf and

https://journals.plos.org/plosone/s/file?id=ba62/PLOSOne_formatting_sample_title_authors_affiliations.pdf

2. Please provide a complete Data Availability Statement in the submission form, ensuring you include all necessary access information or a reason for why you are unable to make your data freely accessible. If your research concerns only data provided within your submission, please write "All data are in the manuscript and/or supporting information files" as your Data Availability Statement.

3. When completing the data availability statement of the submission form, you indicated that you will make your data available on acceptance. We strongly recommend all authors decide on a data sharing plan before acceptance, as the process can be lengthy and hold up publication timelines. Please note that, though access restrictions are acceptable now, your entire data will need to be made freely accessible if your manuscript is accepted for publication. This policy applies to all data except where public deposition would breach compliance with the protocol approved by your research ethics board. If you are unable to adhere to our open data policy, please kindly revise your statement to explain your reasoning and we will seek the editor's input on an exemption. Please be assured that, once you have provided your new statement, the assessment of your exemption will not hold up the peer review process.

4. One of the noted authors is a group or consortium “LIVESAFERS (Leading Innovations for Emergency Support and Vital Enhancements in Resuscitation Simulation) Study Network” In addition to naming the author group, please list the individual authors and affiliations within this group in the acknowledgments section of your manuscript. Please also indicate clearly a lead author for this group along with a contact email address.

5. Your ethics statement should only appear in the Methods section of your manuscript. If your ethics statement is written in any section besides the Methods, please move it to the Methods section and delete it from any other section. Please ensure that your ethics statement is included in your manuscript, as the ethics statement entered into the online submission form will not be published alongside your manuscript.

6. If the reviewer comments include a recommendation to cite specific previously published works, please review and evaluate these publications to determine whether they are relevant and should be cited. There is no requirement to cite these works unless the editor has indicated otherwise.

Additional Editor Comments:

Dear Authors,

Thank you for submitting your protocol on mapping and appraising systematic reviews of resuscitation training. Two peer reviewers have assessed the manuscript; their comments are appended. Having reviewed both the manuscript and their assessments, I am returning it with a decision of MAJOR REVISION.

CRITICAL ISSUE: No Plan for Overlap Assessment

This is an umbrella review and the single most consequential methodological challenge unique to this design is the management of overlapping primary studies across included systematic reviews. When the same underlying trials appear in multiple SRs, double-counting inflates the apparent evidence base and distorts synthesis. This issue is well established in the methodological literature (Abdellatif et al., Trop Med Health, 2025; https://doi.org/10.1186/s41182-025-00764-y).

The current protocol contains no plan to address this. Specifically, it lacks:

1. A primary-study overlap matrix identifying which studies appear across which SRs.

2. Calculation of the Corrected Covered Area (CCA) index, the standard metric for quantifying overlap (slight <5%, moderate 6–10%, high 11–15%, very high >15%).

3. Use a specifically developed and appropriately referenced CCA calculation tool (i.e., GROOVE - Pérez-Bracchiglione et al., Res Synth Methods, 2022; doi:10.1002/jrsm.1557).

4. A pre-specified decision rule for managing overlap contingent on the CCA level found.

This omission is particularly consequential given the manuscript's own purpose: a protocol explicitly designed to hold systematic reviews to a high methodological standard must itself meet that standard. The absence of an overlap plan is a methodological irony that peer reviewers, readers, and citing authors will not overlook.

The revised protocol must address all four points above.

ADDITIONAL ISSUES

1. Search scope: Dedicated SR repositories (Cochrane Library, Epistemonikos, JBI Database, DARE) are absent from the search strategy. These are specifically recommended for umbrella reviews (Abdellatif et al., 2025) and their omission risks missing high-quality SRs not indexed in standard bibliographic databases.

2. GRADE: If GRADE is intentionally excluded because this review focuses on methodological mapping rather than intervention synthesis, this rationale must be stated explicitly in the Methods.

3. Data extraction: The extraction form (Table 2) does not capture the results synthesis method of each included SR. Given the review's methodological focus, this is a relevant variable.

Please submit a revised manuscript with a point-by-point response letter. Acceptance is not guaranteed and will depend on the adequacy of revisions.

Yours sincerely,

Athanasios G. Pantelis, MD, MSc, FMBS, FACS

Acsdemic Editor

PLOS ONE

[Note: HTML markup is below. Please do not edit.]

Reviewer's Responses to Questions

**Comments to the Author**

1. Does the manuscript provide a valid rationale for the proposed study, with clearly identified and justified research questions?

The research question outlined is expected to address a valid academic problem or topic and contribute to the base of knowledge in the field.

Reviewer #1: Yes

Reviewer #2: Yes

**********

2. Is the protocol technically sound and planned in a manner that will lead to a meaningful outcome and allow testing the stated hypotheses?

The manuscript should describe the methods in sufficient detail to prevent undisclosed flexibility in the experimental procedure or analysis pipeline, including sufficient outcome-neutral conditions (e.g. necessary controls, absence of floor or ceiling effects) to test the proposed hypotheses and a statistical power analysis where applicable. As there may be aspects of the methodology and analysis which can only be refined once the work is undertaken, authors should outline potential assumptions and explicitly describe what aspects of the proposed analyses, if any, are exploratory.

Reviewer #1: Yes

Reviewer #2: Yes

**********

3. Is the methodology feasible and described in sufficient detail to allow the work to be replicable?

Descriptions of methods and materials in the protocol should be reported in sufficient detail for another researcher to reproduce all experiments and analyses. The protocol should describe the appropriate controls, sample size calculations, and replication needed to ensure that the data are robust and reproducible.

Reviewer #1: Yes

Reviewer #2: Yes

**********

4. Have the authors described where all data underlying the findings will be made available when the study is complete?

The PLOS Data policy requires authors to make all data underlying the findings described in their manuscript fully available without restriction, with rare exception, at the time of publication. The data should be provided as part of the manuscript or its supporting information, or deposited to a public repository. For example, in addition to summary statistics, the data points behind means, medians and variance measures should be available. If there are restrictions on publicly sharing data—e.g. participant privacy or use of data from a third party—those must be specified.

Reviewer #1: No

Reviewer #2: Yes

**********

5. Is the manuscript presented in an intelligible fashion and written in standard English?

PLOS ONE does not copyedit accepted manuscripts, so the language in submitted articles must be clear, correct, and unambiguous. Any typographical or grammatical errors should be corrected at revision, so please note any specific errors here.

Reviewer #1: Yes

Reviewer #2: Yes

**********

6. Review Comments to the Author

Please use the space provided to explain your answers to the questions above and, if applicable, provide comments about issues authors must address before this protocol can be accepted for publication. You may also include additional comments for the author, including concerns about research or publication ethics.

You may also provide optional suggestions and comments to authors that they might find helpful in planning their study.

(Please upload your review as an attachment if it exceeds 20,000 characters)

Reviewer #1: Comments

I would like to thank the author for selecting this important and timely topic. The focus of the study is highly relevant and addresses significant gaps in the current literature, which adds value to the field.

Line 30 and introduction-The number of systematic reviews on resuscitation training has grown rapidly, offering valuable 32 evidence to guide healthcare decision-making- Please add global evidence of clear guidance on how much it saves lives.

Line 107- include PROSPERO registration number

What is the scope and distribution of published systematic reviews on resuscitation 115 training interventions? Are you considering adult and child neonatal or all?? Pls state

Methodological quality appraisal-will you plan for do piloting??

Limitations-add more limitations different SR on age categories, different methods difficult to analysis

Reviewer #2: This is an Protocol for review of reviews . Clear and sound design. Looking forward for the actual review - study

**********

7. PLOS authors have the option to publish the peer review history of their article (what does this mean?). If published, this will include your full peer review and any attached files.

If you choose “no”, your identity will remain anonymous but your review may still be made public.

**Do you want your identity to be public for this peer review?**  For information about this choice, including consent withdrawal, please see our Privacy Policy.

Reviewer #1: **Yes:**  WDJK Amarasena

Reviewer #2: **Yes:**  Oliver Kalpak MD PhD

**********

[NOTE: If reviewer comments were submitted as an attachment file, they will be attached to this email and accessible via the submission site. Please log into your account, locate the manuscript record, and check for the action link "View Attachments". If this link does not appear, there are no attachment files.]

To ensure your figures meet our technical requirements, please review our figure guidelines: https://journals.plos.org/plosone/s/figures

You may also use PLOS’s free figure tool, NAAS, to help you prepare publication quality figures: https://journals.plos.org/plosone/s/figures#loc-tools-for-figure-preparation.

NAAS will assess whether your figures meet our technical requirements by comparing each figure against our figure specifications.

# Author comment: Author response to Decision Letter 1

**DOI:** 10.1371/journal.pone.0349693.r002

3 May 2026

Please find the attached file for response to reviewers.

Attachment Submitted filename: Response to rev_revDE1_260427.docx

# Editor report: Decision Letter 1

**DOI:** 10.1371/journal.pone.0349693.r003

**Authors:** Athanasios Pantelis, Athanasios Pantelis

4 May 2026

Mapping and Appraising Evidence Syntheses of Resuscitation Training for Healthcare Professionals: Protocol for a Systematic Review of Reviews

PONE-D-25-60471R1

Dear Dr. Wong,

We’re pleased to inform you that your manuscript has been judged scientifically suitable for publication and will be formally accepted for publication once it meets all outstanding technical requirements.

Within one week, you’ll receive an e-mail detailing the required amendments. When these have been addressed, you’ll receive a formal acceptance letter and your manuscript will be scheduled for publication.

An invoice will be generated when your article is formally accepted. Please note, if your institution has a publishing partnership with PLOS and your article meets the relevant criteria, all or part of your publication costs will be covered. Please make sure your user information is up-to-date by logging into Editorial Manager at Editorial Manager® and clicking the ‘Update My Information' link at the top of the page. For questions related to billing, please contact billing support.

If your institution or institutions have a press office, please notify them about your upcoming paper to help maximize its impact. If they’ll be preparing press materials, please inform our press team as soon as possible -- no later than 48 hours after receiving the formal acceptance. Your manuscript will remain under strict press embargo until 2 pm Eastern Time on the date of publication. For more information, please contact onepress@plos.org.

Kind regards,

Athanasios G. Pantelis

Academic Editor

PLOS One

Additional Editor Comments (optional):

Reviewers' comments:

# Editor report: Acceptance letter

**DOI:** 10.1371/journal.pone.0349693.r004

**Authors:** Athanasios Pantelis, Athanasios Pantelis

PONE-D-25-60471R1

PLOS One

Dear Dr. Wong,

I'm pleased to inform you that your manuscript has been deemed suitable for publication in PLOS One. Congratulations! Your manuscript is now being handed over to our production team.

At this stage, our production department will prepare your paper for publication. This includes ensuring the following:

* All references, tables, and figures are properly cited

* All relevant supporting information is included in the manuscript submission,

* There are no issues that prevent the paper from being properly typeset

You will receive further instructions from the production team, including instructions on how to review your proof when it is ready. Please keep in mind that we are working through a large volume of accepted articles, so please give us a few days to review your paper and let you know the next and final steps.

Lastly, if your institution or institutions have a press office, please let them know about your upcoming paper now to help maximize its impact. If they'll be preparing press materials, please inform our press team within the next 48 hours. Your manuscript will remain under strict press embargo until 2 pm Eastern Time on the date of publication. For more information, please contact onepress@plos.org.

You will receive an invoice from PLOS for your publication fee after your manuscript has reached the completed accept phase. If you receive an email requesting payment before acceptance or for any other service, this may be a phishing scheme. Learn how to identify phishing emails and protect your accounts at https://explore.plos.org/phishing.

If we can help with anything else, please email us at customercare@plos.org.

Thank you for submitting your work to PLOS ONE and supporting open access.

Kind regards,

PLOS ONE Editorial Office Staff

on behalf of

Dr. Athanasios G. Pantelis

Academic Editor

PLOS One
